# ER-Associated Degradation of Membrane Proteins in Yeast

**DOI:** 10.1100/tsw.2006.191

**Published:** 2006-08-17

**Authors:** Cédric Pety de Thozée, Michel Ghislain

**Affiliations:** Unité Biochimie Physiologique Institut des Sciences de la Vie Université Catholique de Louvain, B-1348 Louvain-la-Neuve, Belgium

**Keywords:** yeast, ER-associated degradation (ERAD), ubiquitin, proteasome, quality control system, plasma membrane, H^+^-ATPase, ABC transporters

## Abstract

Proteins destined for the secretory pathway are translocated into the endoplasmic reticulum (ER), where they are subjected to a variety of post-translational modifications before they reach their final destination. Newly synthesized proteins that have defect in polypeptide folding or subunit assembly are recognized by quality control systems and eliminated by the 26S proteasome, a cytosolic ATP-dependent proteolytic machinery. Delivery of non-native ER proteins to the proteasome requires retrograde transport across the ER membrane and depends on a protein-unfolding machine consisting of Cdc48p, Ufd1p, and Npl4p. Recent studies in yeast have highlighted the possible function of the Sar1p/COPII machinery in ER-associated degradation of some lumenal and membrane proteins.

## SUBCELLULAR TRAFFICKING OF MEMBRANE PROTEINS

Nascent transmembrane proteins are cotranslationally integrated into the endoplasmic reticulum (ER) membrane through the Sec61 pore complex and subsequent diffusion in the plane of the membrane. Non-ER-resident proteins are then transported to the Golgi apparatus, where sorting to the plasma membrane, vacuoles, and other intracellular compartments occurs (Fig. 1). Intracellular protein transport between the compartments of the secretory pathway is mediated by vesicle carriers that are released from a donor organelle and fused with an appropriate acceptor membrane[1]. Membrane fusion requires small GTPases of the Rab family, which cycle between an active, membrane-associated GTP-bound form on the vesicle and an inactive, soluble GDP-bound form[2]. Activated Rabs recruit effector proteins present on target membranes that tether the two membranes in a loose association. Membrane fusion is then mediated by SNARE (SNAP receptor) and SM (Sec1p in yeast and Munc18 in mammals) proteins[3]. A SNARE protein from one membrane usually interacts with three SNAREs on the other. Conserved domains in the SNAREs reorganize in a zipper-like fashion to form four, long, intertwined helices to provide the energy for membrane fusion. The disassembly of the SNAREs requires Sec17p and Sec18p (NSF in mammals), an ATPase of the AAA (ATPases associated with a variety of cellular activities) family. In addition to their role in SNARE pairing, the SM proteins might prevent unwanted association of SNAREs during their transport back to their respective membranes[4].

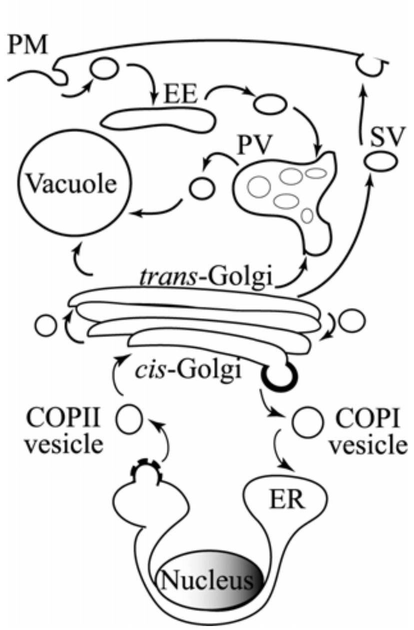

**FIGURE 1.** Schematic overview of the vesicular routes in the secretory and endocytic pathways. The ER is the port of entry of proteins destined for secretion, for the plasma membrane (PM), and for various secretory and endocytic compartments. Correctly folded and assembled cargo proteins are packaged into COPII-coated vesicles and transported to the Golgi network. COPI proteins induce the formation of return vesicles that bring cargo receptors and components of membrane fusion machinery back to the ER. Cargo proteins are transported from the *trans*-Golgi to the plasma membrane via secretory vesicles (SV). Ubiquitin-dependent endocytosis targets some plasma membrane proteins for degradation in the vacuole through early endosomes (EE) and prevacuolar compartments (PV) or multivesicular bodies. Misfolded proteins can be rerouted directly to the vacuole from the Golgi.

Here, we focus on coat protein (COP) complex II–dependent ER export and COPI-mediated retrieval, as these highly selective processes contribute to quality control and the ER-associated degradation (ERAD) of non-native proteins.

## COPII-Dependent ER Export

Newly synthesized membrane and secretory proteins are sorted from ER-resident proteins and packaged into COPII–coated vesicles[5]. Cytosolic Sar1p-GDP is converted to a membrane-embedded Sar1p∙GTP form through transient binding to Sec12p, an ER membrane GDP/GTP exchange factor (GEF) (Fig. 2). Sar1p∙GTP recruits the heterodimeric Sec23/24p complex to form a prebudding complex, which sorts membrane and soluble cargo from resident proteins and concentrates them in the bud. The soluble cargo proteins bind to various cargo receptors that associate with Sec23/24p. Three binding sites in Sec24p have been mapped by site-directed mutagenesis[6]. One is implicated in the capture of the SNARE protein, Bet1p[7], and transmembrane cargo proteins, such as the Yor1 ABC transporter, through the C-terminal DxE sorting signal[8]. The prebudding complex binds Sec13p and Sec31p, thereby promoting the formation of a scaffold complex, which causes the vesicle to bud out from the ER membrane. Vesicle scission then leads to the dissociation of Sec12p from Sar1p∙GTP and subsequent GTP hydrolysis, which causes Sar1p-GDP and the coat subunits to dissociate from the vesicle membrane. It has been suggested that GTP hydrolysis by Sar1p promotes the exclusion of aberrant proteins from COPII vesicles[9].

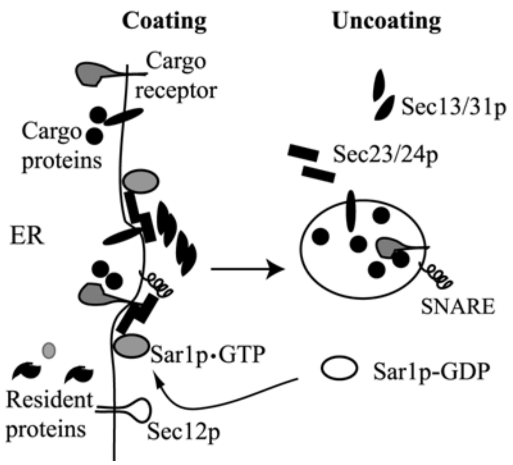

**FIGURE 2.** Assembly and disassembly of COPII vesicles. The small G protein, Sar1p, in the cytoplasm is bound to GDP, which is then replaced by GTP, a process requiring the transmembrane guanine nucleotide exchange factor, Sec12p. Sar1p•GTP (grey oval) is then recruited at the cytosolic side of the ER membrane. Two large complexes, Sec23/24p and Sec13/31p, then bind sequentially to form a prebudding complex, which pinches the membrane into a vesicle. Sec24p interacts directly with membrane cargo proteins. The interaction with soluble cargos requires specific receptors. The release of COPII vesicles from the ER membrane dissociates Sec12p from Sar1p•GTP, activating its GTPase activity. GTP hydrolysis results in Sar1p-GDP/COPII uncoating of the vesicle.

Various subtypes of COPII-coated vesicles adapted to different cargo proteins have been reviewed recently[10]. Sec12p, Sar1p, and the Sec23p/Sec24p complex are also required for the formation of autophagosomes[11].

### Retrograde Transport to the ER

Protein retrieval from the Golgi to the ER is mediated by the COP complex I, composed of subunits α, β, β', γ, δ, ε, and ξ[12]. Assembly of the COPI coat at the cytoplasmic side of the *cis*-Golgi membrane is initiated by the binding of Arf1/2p•GTP[13]. The cargo proteins are recruited into the budding COPI-coated vesicles, which are then released from the Golgi membrane and transported to the ER[14]. Of the four GEFs that can act on Arf1/2p–GDP, only Gea1p and Gea2p function in retrograde transport from the Golgi to the ER[15].

## QUALITY CONTROL SYSTEMS

### Quality Control in the ER

To enter the secretory pathway, proteins must be correctly folded and, in some cases, glycosylated or assembled into multiprotein complexes[16,17,18]. The folding of newly synthesized proteins into their proper conformation involves ER-resident molecular chaperones, such as soluble Lhs1p[19] or Kar2p, the homolog of mammalian BiP[20,21], and membrane-bound Pbn1p[22]. Protein disulfide isomerases or oxidoreductases, including Pdi1p, four other nonessential Pdi1-related proteins[23], and Ero1p[24], ensure that irreversible formation of disulfide bonds does not occur until protein folding is complete. In yeast, it is not known which of the several peptidyl-prolyl isomerases are responsible for protein folding in the ER[25].

The ER has a quality-control machinery that recognizes non-native proteins on the basis of their modified thermodynamic stability[26] and ensures that only native proteins are transported along the secretory pathway. As folding sensors, the ER lumenal chaperones and oxidoreductases Pdi1p[27] and Esp1p[28] are key components of this machinery. *N*-linked oligosaccharides in non-native glycoproteins are recognized by specific lectin chaperones. In yeast, these are Cne1p[29,30], membrane-bound Mnl1p (alias Htm1p)[31,32], and lumenal Yos9p, which shows similarities with mannose-6-phosphate receptors[33,34], the corresponding mammalian equivalents being calnexin, EDEM (ER degradation enhancing α-mannosidase-like protein), and OS-9, which is overexpressed in human osteosarcomas. Unlike Mns1p, Mnl1p is not a processing mannosidase, but a lectin that recognizes the Man8GlcNAc2 oligosaccharides that serve as the signal targeting mutant vacuolar CPY* (carboxypeptidase Y) and plasma membrane Pdr5* (multidrug ABC-transporter) for ERAD[32]. According to the mannose timer hypothesis, a Man8 structure on a misfolded protein signals that the polypeptide has resided in the ER for some time without acquiring its native structure[16,17].

Kar2p, Pdi1p, Eps1p, and Mnl1p are also involved in ERAD by remodeling the aberrant proteins into a partially unfolded or reduced form, more amenable for retrotranslocation, or dislocation[35]. Membrane proteins with misfolded segments exposed at the cytosolic side of the ER membrane require additional folding sensors for quality control. The role of the cytosolic protein chaperones/cochaperones and ER-associated protein ubiquitin ligase is discussed in the section dealing with the ERAD pathway.

## Quality Control in the Golgi Network

When the quality-control system in the ER is saturated, for instance, in stress conditions, the lumenal misfolded proteins that are no longer retained in the ER have to be recognized by post-ER quality-control mechanisms and either transported back to the ER for ERAD or targeted for vacuolar degradation[36]. Post-ER quality control may also be required for the recognition of certain protein conformational lesions not recognized by the ER quality-control system. This hypothesis is based on the behavior of the Pma1-7 variant of the plasma membrane $H^+$-ATPase, which has one mutation in the central cytosolic loop and a second at an extracytoplasmic junction between two transmembrane segments. Pma1-7p is targeted for vacuolar degradation through the Golgi, instead of being degraded through the ER pathway[37]. In the Golgi apparatus, the vacuolar protein sorting receptor, Vps10, plays a dual role, as it also targets soluble misfolded proteins to the vacuole[38,39]. Moreover, vacuolar targeting of membrane cargo proteins that contain aberrant membrane domains or are not completely oligomerized is mediated by a membrane ubiquitin-ligase, Tul1p[40]. Although it mediates ubiquitin-dependent endocytosis of several plasma membrane transporters[41,42,43] and the sorting of the general amino acid permease, Gap1p, from the Golgi to the multivesicular bodies prior to vacuolar degradation[44], another HECT-domain E3, Rsp5p, is needed for degradation of overexpressed ERAD substrates[45].

The requirement for ER-to-Golgi transport for the proteasomal degradation of several misfolded proteins is suggested by the stabilization effects of mutations impairing vesicular transport (*sec18*) or the Sar1p/COPII machinery (*sec12, sec23*) (Fig. 3). Erv29p, which appears to function as a cargo receptor for ER export, is required for efficient degradation of misfolded secretory proteins, apparently by delivering them to the Golgi[46]. The COPII vesicles also contain a GPI inositol deacylase, Bst1p, which is specifically involved in the transport of misfolded cargo proteins to the Golgi[47]. However, several misfolded membrane proteins do not require ER-to-Golgi transport for their degradation. Soluble ERAD substrates may therefore require additional quality control in post-ER compartments[46]. Alternatively, ER degradation could be confined to certain ER subcompartments, which are reached by soluble proteins by vesicular transport through the Golgi network[47]. However, the possibility that a defect in ER-to-Golgi transport leads to a overall perturbation of the ER cannot be totally excluded[48]. Whichever hypothesis is correct, it remains to be elucidated how soluble ERAD substrates are recognized in the Golgi and transported back to the ER. As a mutation in Sec12 COPI subunit has a slight stabilization effect[47], there must be a different retrograde transport mechanism.

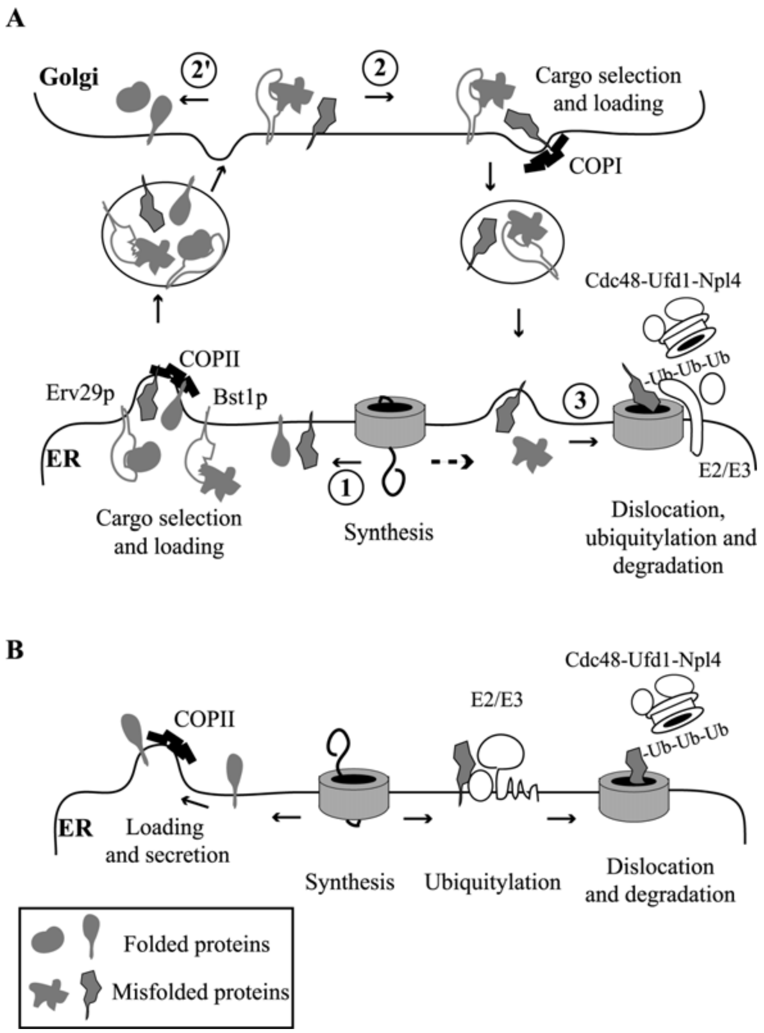

**FIGURE 3.** Distinct ERAD pathways according to membrane topology of misfolded domains. (A) Newly synthesized secretory and membrane proteins are packaged into COPII vesicles (1). Soluble proteins bind to cargo receptors, such as Bst1p and Erv29p, whereas membrane proteins may interact directly with components of the coatomer. In the Golgi, proteins containing lumenal lesions are sorted out and transported back to the ER through COPI vesicles or another unidentified retrograde transport mechanism (2). The misfolded proteins are transported through the Sec61 translocon or Sec61-like channels to the cytosol, where they are ubiquitylated by ER ubiquitin-conjugating enzymes (E2) and ligases (E3). The Cdc48p/Ufd1p/Npl4p complex binds to ubiquitylated proteins and transfer them to the proteasome (3). Note that some misfolded proteins are directly targeted for dislocation, bypassing ER-Golgi traffic (broken arrow). (B) Transmembrane proteins containing cytosolic lesions are retained in the ER as a consequence of their ubiquitylation by a second E2/E3 machinery. After dislocation from the ER to the cytosol, ubiquitylated substrates are delivered to the proteasome by the Cdc48p/Ufd1p/Npl4p complex.

## THE UNFOLDED PROTEIN RESPONSE

The accumulation of unfolded proteins in the lumen of the ER induces a coordinated adaptive program called the unfolded protein response, or UPR[49]. The UPR alleviates stress by up-regulating protein folding and degradation pathways in the ER and inhibiting protein synthesis[50,51,52]. The UPR is induced by tunicamycin, an inhibitor of *N*-linked glycosylation, disruption of ER-associated degradation, and overexpression of ERAD substrates[53,54]. In the presence of high levels of unfolded/misfolded proteins, the Kar2p/Ire1p complex dissociates, triggering activation of Ire1p[55]. This ER transmembrane kinase/endonuclease is able to process the pre-mRNA of the *HAC1* gene into a mature transcript. Hac1p synthesis then leads to transactivation of target genes through binding to a conserved Unfolded Protein Response Element (UPRE) in the promoter region[56]. The target genes encode ER chaperones (e.g., Kar2p) and components of the ubiquitin system (e.g., Ubc7p, Hrd1p, and Tul1p), exocytic pathway (e.g., Sec12p), and phospholipid biosynthetic pathways (e.g., Lcb1p). Thus, ERAD, vesicular process, and maintenance of the ER membrane are tightly coordinated processes[57,58].

Deletion of the *IRE1* gene impairs the degradation of soluble (CPY*) or membrane-bound (mouse MHC class-I heavy-chain H-2$K^b$) ERAD substrates. This defect in degradation is suppressed by constitutively inducing the UPR via expression of Hacp[54]. However, UPR induction is not required for the degradation of some other membrane ERAD substrates, such as Ste6-Q1249X (Ste6*p)[59].

## ACCUMULATION OF ER-DERIVED COMPARTMENTS

A second means of coping with non-native proteins in the ER is to sequester them in special subcompartments. When expressed in yeast, plant plasma membrane $H^+$-ATPase (PMA) isoforms induce the formation of stacked membrane pairs surrounding the nucleus envelope[60]. These are known as karmellae and were originally identified in cells overexpressing the ER membrane 3-hydroxy 3-methylglutary coenzyme A (HMG-CoA) reductase[61]. A second type of ER subcompartment is known as the ER-associated compartment (ERAC), which consists of a network of tubulovesicular structures[59]. ERAC formation is induced by specific variants of yeast membrane transporters, such as Pma1-D378N[62] and the truncated ABC transporter, Ste6-Q1249X[63], as well as by overexpression of Pma2, an $H^+$-ATPase isoform normally expressed at low levels[64].

Resident ER lumenal proteins, such as Kar2p, are detected in ERACs, showing that these are connected to the ER. In cells expressing the truncated Ste6-L1239X variant, the presence of ERACs does not adversely affect the secretory pathway and the UPR is not induced[59]. ERAC induction by Ste6-G38D, another variant mutated in the first transmembrane segment, is not impaired by mutations in *SEC18* and *SEC23*, indicating that ER-to-Golgi transport is not required[59]. Strikingly, the accumulation in ERACs of heterologously expressed CFTR (cystic fibrosis transmembrane conductance regulator), a mammalian homolog of Ste6p, does require the Sar1p/COPII machinery[65]. Density gradient centrifugation showed that ERACs and karmellae sediment at the same buoyant density[66] and probably represent different states of the same initial ER subcompartment. The Pma1-D378N variant is misfolded, as shown by its increased sensitivity to limited trypsin digestion[67]. In purified ERACs, it has no ATPase activity, indicating that its accumulation in ERACs fails to restore the wild-type conformation, despite the presence of Kar2p (A. Goffeau, personal communication). Pma1-G381A is another variant that accumulates in Kar2p-containing ERACs; however, it eventually reaches the plasma membrane and is then targeted to the vacuole for degradation[68].

## ER-ASSOCIATED DEGRADATION

Non-native proteins in the ER are targeted for degradation by the 26S proteasome. In the ERAD pathway, ubiquitin has a role distinct from its roles in the internalization of plasma membrane proteins targeted for vacuolar degradation and in protein sorting from the Golgi to the vacuole[69,70]. Here, we summarize the relevant components of the ubiquitin-proteasome system[18,71].

## The Ubiquitin-Proteasome System

Proteins are marked for degradation by post-translational conjugation to ubiquitin, a 76-amino acid protein that is highly conserved between man and yeast[72]. ATP-dependent activation of ubiquitin is catalyzed by Uba1p, a ubiquitin-activating enzyme, or E1. The C-terminus of activated ubiquitin is then covalently linked to the ε-amino group of a lysine residue on the target protein via the sequential actions of ubiquitin-conjugating enzymes (Ubcs or E2s) and specific ubiquitin-protein ligases (E3s)[73,74]. In most cases, ERAD substrates form multiubiquitylated conjugates, as the Lys48 residue of ubiquitin can be conjugated to another ubiquitin molecule[75].

The 26S proteasome is a large multisubunit complex (Mr = 2 MDa) composed of two outer 19S caps and one inner 20S catalytic core[76]. The 19S cap is involved in the recognition, binding, and unfolding of ubiquitylated proteins. It consists of a lid (Rpn subunits 3, 5-9, and 11-12) and a base (AAA-ATPases Rpt1-6 and Rpn subunits 1-2), which contacts the α-subunits of the 20S core. The cap is linked to the base through the multiubiquitin-binding protein, Rpn10. The catalytic core has two α outer and two β inner rings, each composed of seven distinct subunits (Pre1-10p, Pup1-3p, and Scl1p). The outer rings have no proteolytic activity and may play a structural role. In contrast, three of the subunits making up the inner rings are proteases cleaving after acidic, basic (trypsin-like), and hydrophobic (chymotrypsin-like) residues[77].

Multiubiquitylated substrates bind to the ubiquitin-associated (UBA) domains of Rad23p and Dsk2p. These proteins also contain one ubiquitin-like (UBL) domain that is recognized by the proteasome subunits, Rpn1p and Rpn2p. Based on these properties, Rad23p and Dsk2p could escort multiubiquitylated substrates from ubiquitin-protein ligases to the proteasome[78]. Rad23p also mediates the binding to the proteasome of Png1p, a peptide:N-glycanase that discriminates between correctly folded and misfolded glycoproteins[79]. In cells expressing CPY*, immunoprecipitation of CPY* results in coimmunoprecipitation of Cdc48p and Png1p[80]. These two results support the view that Png1p plays a role in the proteasome-dependent degradation of non-native glycoproteins that have been extracted from the ER membrane.

## The Cdc48/Ufd1/Npl4 Complex

Proteasomal degradation of ER secretory and transmembrane proteins requires retrograde transport out of the ER back to the cytosol. Whether this occurs through the Sec61 translocon[81] or requires an alternative channel consisting of Der1p[82,83] or ubiquitin-protein ligases, Hrd1p[84,85] and Doa10p[86], is still a matter of debate[71,87]. Ubiquitylated ERAD substrates are released from the ER by a ubiquitin-specific chaperone complex consisting of Cdc48p, Ufd1p, and Npl4p[88,89,90,91,92,93], where ubiquitin chains are further extended by Ufd2p, an E4 multiubiquitylation enzyme[94]. Rad23p (or Dsk2p) binds to the multiubiquitin chains and delivers the multiubiquitylated substrates to the proteasome for degradation[95,96].

Like Sec18p, Cdc48p (p97 or valosin-containing protein in mammals) is an AAA-ATPase, which has a duplication of the ATP-binding domain and the SRH conserved motif[97]. X-ray crystallographic studies[98,99] showed six subunits assembled into a ring, the overall shape of which changes during the ATPase catalytic cycle. A nucleotide-dependent conformational switch may apply tension to bound proteins and, thereby, allow polypeptide unfolding. Cdc48p binds to the proteasome and has a high affinity for the tetraubiquitin chain[100]. Mammalian p97 is directed to homotypic membrane fusion via binding to a Ubx domain-containing protein, p47, which inhibits ATPase activity *in vitro*[101,102]. The yeast homologue of p47, Ubx1p (alias Shp1), and six other Ubx proteins associate with Cdc48p via their Ubx domain, which shows a high structural similarity to ubiquitin. These proteins are required for the degradation of engineered soluble ubiquitin protein fusions[103,104] and CPY*[105,106]. Ubx2p is an integral ER membrane protein that recruits the Cdc48p/Ufd1p/Npl4p complex to the ubiquitin ligases, Doa10p and Hrd1p, and to ERAD substrates[105,106]. Ubx2p contains a N-terminal UBA domain, which is crucial for ERAD, but dispensable for the degradation of soluble cytoplasmic or nuclear proteins. This result may indicate that binding of ubiquitylated ER proteins to the UBA domain of Ubx2p is required for their dislocation[105]. Loss of Ubx2p leads to a slight decrease in the amount of membrane-bound Cdc48p, indicating the presence of additional membrane-recruitment factors for the Cdc48p/Ufd1p/Npl4p complex[105,106]. Ubx2p also interacts with two putative dislocation pore proteins, Der1p and Dfm1p[83], probably via the ER ubiquitin ligases[106]. Taken together, these data suggest a pivotal role for Ubx2p in promoting dislocation of ERAD substrates from the ER to the cytosol[106]. According to another model speculating that Der1p might be the primary Cdc48p receptor in the ER, Ubx2p mediates the transfer of ubiquitylated proteins from the ubiquitin ligases to the Cdc48p/Ufd1p/Npl4p complex[107].

Ufd1p was identified by screening for mutants with an impaired ubiquitin fusion degradation (UFD) pathway[108]. Its N-domain has striking similarities with that of Cdc48p. The NMR structure shows two distinct binding sites for mono- and polyubiquitin molecules[109], so Ufd1p may also bind to Ubx2p. Mutants in the *NPL4* gene show an altered nuclear envelope morphology and nuclear pore function[110]. Degradation of cytosolic proteins is normal in the *npl4* mutants. However, they are impaired in degradation of ER proteins, such as HMG-CoA reductase, which is stabilized in a polyubiquitylated form[88]. These results indicates that Npl4p plays an intermediate role between ERAD substrate ubiquitylation and proteasomal degradation. Binding of the Npl4p/Ufd1p heterodimer to Cdc48p is partly mediated by a ubiquitin-fold domain (UBD) in Npl4p[90,111].

Binding of Ufd2p to Cdc48p is inhibited by the WD40 repeat protein, Doa1p (alias Ufd3p), which utilizes the same docking site on Cdc48p[94]. Doa1p is required for the maintenance of cytosolic-free ubiquitin pools[108,112]. Doa1p and Otu1p, a deubiquitylation enzyme, bind to Cdc48p simultaneously, enhancing their inhibitory effect on Ufd2p[94]. On the other hand, the C-terminal end of Doa1p interacts with the Ubx domain[103]. It is therefore likely that the binding of Ubx2p to Cdc48p can also be inhibited by Doa1p. In conclusion, the multiubiquitylated vs. deubiquitylated state of an ERAD substrate and its subsequent targeting to the proteasome for degradation are determined by a subtle balance between the distinct Cdc48p cofactors[94].

## Degradation Determinants in Short-Lived ER-Resident Proteins

Apart from its role in protein quality control, the ERAD pathway targets specific ER proteins for proteasome-mediated degradation or maturation. Regulated degradation is often mediated by specific recognition of small regions of the target sequence, named degrons[113], whereas quality-control mechanisms recognize common structural hallmarks of non-native proteins. It is therefore crucial to identify the structural determinants that target normal ER-resident protein for proteasome-mediated degradation. The following examples of regulatory degradation concern two proteins involved in lipid biosynthesis.

Mevalonate is used as a precursor of activated isoprenes and sterols. Its synthesis is catalyzed by HMG-CoA reductase. The yeast Hmg2p isoform is an ER-resident membrane protein, consisting of eight transmembrane segments and a catalytic C-terminal domain exposed at the cytosolic side of the membrane. On accumulation of sterols and other isoprenoids, Hmg2p undergoes rapid ER-associated degradation[84]. Hmg2p ubiquitylation is mediated by the Hrd1p/Hrd3p E3 complex and is positively regulated by farnesyl pyrophosphate. High levels of farnesyl pyrophosphate may induce structural changes over the entire transmembrane domain so that Hmg2p is recognized by the ER quality-control system as an ERAD substrate. The recognition mechanisms involve structural features distributed over the membrane domain, rather than primary sequence motifs[114]. Thus, the distributed degron in Hmg2p combines aspects of regulation and quality control[115].

The ER-associated $\Delta$-9 fatty acid desaturase, Ole1p, converts palmitoyl (16:0) and stearoyl (18:0) CoA to palmitoleic (16:1) and oleic (18:1) acids, thereby regulating unsaturated fatty acid pools and membrane fluidity[116]. Ole1p is a short-lived protein that is degraded through the ERAD pathway[91].

Unsaturated fatty acids repress Ole1p expression. This regulation is mediated by two NF-κB-related transcription factors, Mga2p and Spt23p[117], which are initially synthesized as inactive precursors.[90,91]. The dimeric Spt23 complex is anchored in the ER membrane via a C-terminal membrane spanning segment. Spt23p activation is triggered by Rsp5-dependent ubiquitylation of one subunit and subsequent cleavage by the proteasome. The processed, transcriptionally active domain is released from the unmodified partner by the activity of the Cdc48p/Ufd1p/Npl4p complex[90,118,119]. Rsp5p is not required for Mga2p processing, but is required for its release from the ER membrane by promoting ubiquitylation and proteasomal degradation of the intact partner[120]. It remains to be determined whether Doa10p is eventually involved in this process and, if not, to identify the molecular mechanisms that target Spt23p for Rsp5p-mediated ubiquitylation.

## The ERAD Pathway for Mutated Secretory Proteins

Several model proteins have been used to identify the different components of the ERAD pathways (Fig. 4). CPY* is a prototypal lumenal ERAD substrate. This mutated version of vacuolar carboxypeptidase has a Gly to Arg substitution near the active site serine. In contrast to wild-type CPY, CPY* is rapidly degraded by trypsin *in vitro*, indicating an altered structure. No Golgi-specific carbohydrate modification has been detected on CPY*[121].

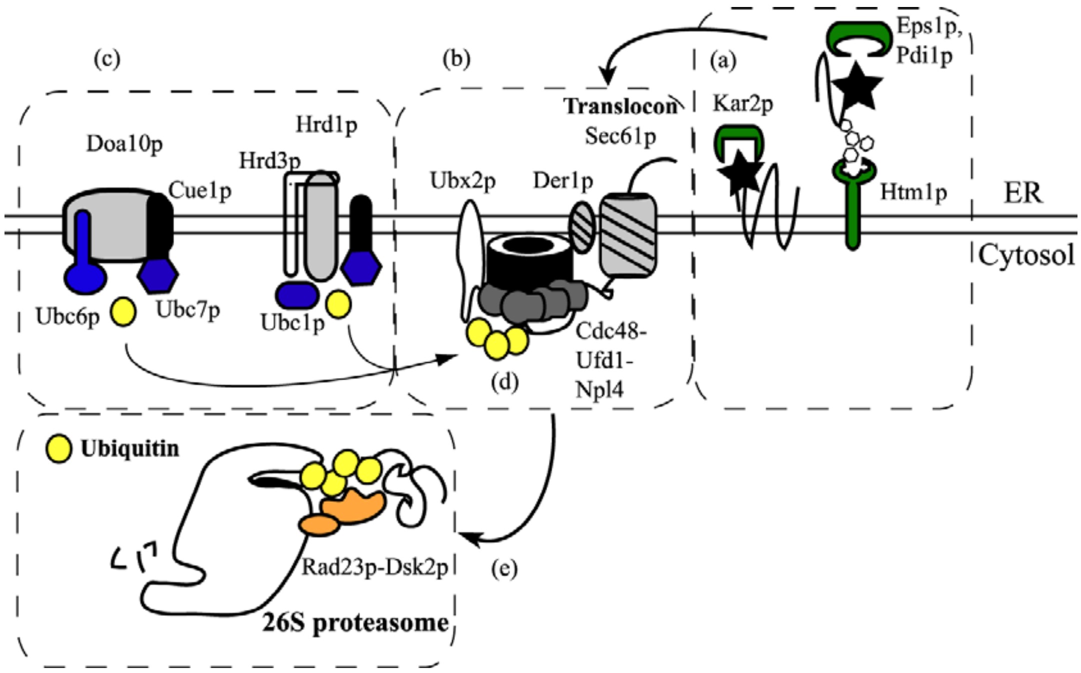

**FIGURE 4.** Proteasome-mediated degradation of non-native ER proteins. The molecular chaperone, Kar2p, the oxidoreductases, Pdi1p and Esp1p, and the lectin, Htm1p, capture misfolded lumenal domains (black star) in the ER via, respectively, exposed hydrophobic surfaces, unbound cysteine residues, and non-native *N*-glycans (a) .The partially unfolded proteins are targeted for dislocation through the Sec61p pore complex or Der1p (b). While emerging from the ER, the ERAD substrates are multiubiquitylated by Ubc7p and, to a lesser extent, Ubc1p, acting in concert with the protein ligase, Hrd1p. Ubc7p is anchored in the ER membrane through Cue1p, and Hrd3p protects Hrd1p from self-ubiquitylation (c). Ubx2p binds to the ubiquitylated polypeptides and transfers them to the Cdc48p/Ufd1p/Npl4p complex (d). Finally the multiubiquitylated polypeptides are delivered to the 26S proteasome by an escort complex consisting of Rad23p and Dsk2p and degraded (e). Ubiquitylation of membrane proteins with a cytosolic lesion (not shown) is preferentially catalyzed by Ubc7p, Ubc6p, and Doa10p.

Cells mutated in the Sar1p/COPII machinery (*sec12*, *sec23*) or ER-to-Golgi transport (*sec18*, *ufe1*, *sed5*, *erv29*) show impaired CPY* degradation[46,47,48,122]. Ufe1p and Sed5p are SNAREs of the ER and Golgi, respectively, whereas Erv29p is involved in loading certain cargo proteins into COPII vesicles (see Fig. 3). These results suggest that Golgi quality-control mechanisms are required for CPY* degradation[46]. Alternatively, ER degradation may be confined to specialized ER subcompartments that are reached by soluble proteins by retrograde transport from the Golgi[47]. However, CPY* degradation is only slightly decreased by the *sec21* or *sec27* mutations, which impair retrograde transport from the Golgi to the ER[47,48]. On the other hand, the stabilization of CPY* in cells with an impaired early secretory pathway may result from morphological disturbance of the ER and/or mislocalization of Kar2p[123,124].

Retrograde transport of CPY* through the Sec61 translocon depends on Kar2p, Der1p[82], and different ER luminal DnaJ chaperone homologues, including Sec63p[81], Scj1p, and Jem1p[125]. Ubiquitylation of CPY* is catalyzed by Ubc7p, Ubc6p[126], and Ubc1p[53]; Ubc7p being the major E2 of the ERAD pathway. Ubc1p and Ubc7p act in concert with Hrd1p (alias Der3p) ubiquitin ligase[53,84,127]. The E3 protein is protected from self-ubiquitylation by its interaction with a second ER membrane protein, Hrd3p[128]. Substrate binding to Hrd3p might trigger Hrd1p activation through its release from the Hrd1p/Hrd3p complex. The Cdc48/Npl4/Ufd1 protein complex binds to multiubiquitylated CPY* and transfers it from the ER to the 26S proteasome[92].

## The ERAD Pathway for Mutated Transmembrane Proteins

The pathways that target non-native membrane proteins for proteasome-mediated degradation are different depending on the lumenal or cytosolic localization of the lesion.

### *ER-Associated Degradation of Transmembrane Proteins with a Cytoplasmic Lesion*

#### Plasma Membrane ABC Transporter

Ste6p is an ATP-binding cassette (ABC) transporter that functions at the plasma membrane to export the mating pheromone, **a**-factor, from cells. It is composed of two homologous halves, each consisting of six transmembrane domains and a cytosolic nucleotide-binding domain[129]. Step6 is slowly targeted for degradation in the vacuole through ubiquitin-mediated endocytosis[130]. However, Ste6-Q1249-X (Ste6*) lacking the final 52 amino acids in the C-terminal cytosolic tail is rapidly degraded through the ERAD pathway with a half-life of 10 min[63].

ER-associated degradation of Ste6p* does not require ER-to-Golgi transport, as the loss of Sec12p and Sec18p has no effect[131]. This supports the notion that a membrane ERAD substrate with a cytosolic lesion is retained in the ER (see Fig. 3B), consistent with the formation of ERACs. Kar2p is dispensable, whereas the cytosolic Hsp70 chaperone, Ssa1p, and the Hsp40 cochaperones, Ydj1p and Hlj1p, are not[131]. Ydj1p, a known cochaperone for Ssa1/2p, is tethered to the ER membrane by prenylation, and Hlj1p is a C-terminally anchored ER membrane protein. Ubiquitylation of Ste6p* is mediated by ubiquitin ligase Doa10p[131]. This E3 enzyme associates preferentially with Ubc6p and Ubc7p, either directly or indirectly through the Cue1 ER-anchor protein[86]. Doa10p can be partially replaced by the second ER ubiquitin ligase, Hrd1p/Der3p; both E3s contain a RING-H2 (for Really Interesting New Gene) zinc finger domain, which mediates the transfer of ubiquitin from E2s to the target protein[131]. Doa10p is also required for the ER-associated degradation of the soluble Mat$\alpha$2 transcriptional repressor[86]. Ste6p* degradation does not require the Sec61 translocon pore, but, as shown for CPY*, could depend on the Sec61 homologue, Ssh1p. These results must be treated with caution, as they depend on the growth conditions and strains used[131].

### Plasma Membrane $H^+$-ATPase

Pma1p is a stable plasma membrane ATPase of the P-type family that pumps protons out of the cell at the expense of ATP hydrolysis. The generated proton chemical gradient drives the uptake of nutrients and ions. The 100-kDa polypeptide consists of two large cytosolic loops and ten membrane-spanning segments, and both the N- and C-termini are exposed at the cytosolic side of the membrane[66]. The Pma1-D378N variant is poorly folded, as judged by its extreme sensitivity to trypsin, and accumulates in ERACs prior to ER-associated degradation[62,67]. Unlike the intact pump, Pma1-D378N binds Esp1p, an ER membrane protein disulfide isomerase that functions in quality control. The loss of Esp1p allows Pma1-D378N to travel to the plasma membrane[28]. No stabilization of Ste6p is seen in cells lacking Esp1p, indicating that Eps1p is a specific recognition factor[131].

## *ER-Associated Degradation of Transmembrane Proteins with a Lumenal Lesion*

### Plasma Membrane ABC Transporter Pdr5

The multidrug transporter, Pdr5p, is another plasma membrane protein of the ABC family and consists of 12 membrane-spanning segments and two ATP binding domains[132,133]. Compared to Ste6p, Pdr5p shows a reverse sequential order of the membrane and cytosolic domains. The mutant form Pdr5-C1427Y (or Pdr5*) is retained in the ER membrane[134] and is degraded by the ERAD pathway with a half-life of 18 min[127]. There is no direct evidence that it is misfolded, although its glycosylation pattern differs from that of intact Pdr5p[127]. It has been proposed that replacement of the cysteine residue affects the formation of disulfide bonds with other cysteine residues facing the ER lumen[134].

The Hrd1p/Hrd3p complex is required for ubiquitylation of Pdr5*, as shown for lumenal CPY*. The role of the second ER ubiquitin-protein ligase, Doa10p, remains to be tested. Dislocation of the mutated ABC transporter into the cytosol is mediated by the Sec61 channel independently of Der1p. It has been proposed that Pdr5* most likely enters the translocon via a lateral gating event within the membrane[127]. The molecular chaperone, Htm1p, but not Kar2p, is involved in the degradation process[32,127,135].

### Engineered Membrane Chimeras with Misfolded Lumenal Domain

Wsc1p is a signaling protein anchored to the plasma membrane by a single transmembrane span[136]. The *N*-terminal extracellular domain was replaced with domain KHN (a Kar2 signal peptide fused to the simian virus 5 HA-Neuraminidase ectodomain[47]) to generate a membrane chimeric protein with a misfolded ER lumenal domain[122]. The resulting protein fusion, KWW, is degraded with a half-life of 35 min. KWW degradation depends on ER-resident Htm1p, Cue1p, Der1p, and Hrd1p, but not on Doa10p[122] (see Fig. 4). The stabilization effect of *sec12* and *sec18* mutations indicates that KWW degradation also requires ER-to-Golgi transport, as shown for misfolded secretory proteins[122]. This result was the first evidence that membrane ERAD substrates may cycle between the ER and Golgi (see Fig. 3).

Comparable results have been reported for the CTG* chimera, consisting of CPY* fused to GFP via a transmembrane segment from Pdr5p[137]. In addition, proteasomal degradation of CTG* is dependent on Dsk2p and Rad23p[138]. However, in contrast to the results obtained with KWW[122], CTG* degradation is independent of Der1p, consistent with the behavior of Pdr5*, which is degraded in cells lacking Der1p[127]. Another difference with the results reported in[122] is that CTG* degradation does not require ER-Golgi cycling, as CTG* is degraded in the *ufe1-1* mutant in which ER-to-Golgi transport is impaired. CTG* degradation is independent of Kar2p, indicating that recognition of the unfolded CPY* moiety in the ER lumen is not the primary step targeting the protein chimera for ERAD[137]. As CTG* is stabilized in cells lacking the cytosolic Hsp70 chaperone, Ssa1p, or the Hsp40 proteins, Hlj1p, Cwc23p, and Jid1p, it is possible that the GFP moiety in CTG* activates a cytosolic quality control system[137]. As GFP is stable in yeast cells, another possibility is that the misfolded domain of CPY* in the CTG* construct is responsible for a signaling event that recruits the Ssa protein machinery onto the GFP moiety of CTG* for unfolding of this domain to occur.

### *Transmembrane Proteins Exposing Misfolded Domains to the ER Lumen and Cytosol*

The numerous studies summarized so far indicate that membrane proteins are targeted for proteasomal degradation through distinct pathways, depending on the localization of the lesion. What is the fate of a membrane protein having two lesions, one exposed at the ER lumen and the other to the cytosol? This question was addressed by analyzing the fate of the KSS chimera, consisting of Ste6p* (with a cytosolic misfolded domain) fused to the KHN domain (acting as an ER lumenal misfolded domain). KSS degradation was no longer dependent on functional ER-to-Golgi transport or on Der1p, supporting the view that the ERAD substrate is retained in the ER[122]. KSS is degraded as efficiently in *htm1Δ* cells as in wild type cells, even though the chimera contains the misfolded lumenal KHN moiety that renders KWW degradation dependent on Htm1p[122]. Moreover, KSS ubiquitylation is only mediated by Doa10p, as is the case for Ste6p* alone[122,131].

## CONCLUDING REMARKS

The use of modular ERAD substrates has shown that newly synthesized ER-transmembrane proteins are monitored by a two-step quality-control system. The first checkpoint is located at the cytosolic face of the membrane. If a lesion is detected, the membrane protein is ubiquitylated and degraded by the proteasome. This step predominates over the second checkpoint that monitors domains localized in the ER lumen and targets misfolded proteins for proteasomal degradation through a pathway shared by soluble secretory proteins. Whether this second pathway requires ER-to-Golgi transport is still controversial. Strikingly, mutations blocking COPI-mediated retrograde transport to the ER have a slight effect on ER-associated degradation. In yeast degradation of the cystic fibrosis transmembrane conductance regulator (CFTR), a mammalian ABC transporter homologous to yeast Pdr5p and Ste6p implicates the Sar1p/COPII machinery, independently of its function in ER export[65]. Heterologously expressed CFTR is sequestered into Kar2p-containing ER subcompartments before degradation through the ERAD pathway[139,140]. The sequestration and degradation of the EGFP-CFTR chimera are impaired by mutations in the Sar1p/COPII machinery (*sec12*, *sec1*3, and *sec2*3; all target genes of the UPR), but not by the *sec18-1*$^{ts}$ mutation, indicating that the function of the Sar1p/COPII machinery does not require vesicular fusion[65]. These results raise the possibility that all newly synthesized proteins are subjected to a Sar1p/COPII sorting mechanism, irrespective of their ultimate secretory or degradation fate. In mammals, however, CFTR degradation does not require the COPII machinery[141]. Further experiments will be needed to address these controversial issues and to dissect the sorting mechanisms involved in the ER and post-ER compartments.

## ACKNOWLEDGMENTS

We acknowledge the financial support from the Interuniversity Poles of Attraction Programme (Belgian State, Prime Minister’s Office, Federal Office for Scientific, Technical and Cultural Affairs) and from the Belgian National Fund for Scientific Research. Cédric Pety de Thozee was the recipient of a FRIA doctoral fellowship.
